# Healthcare workers’ knowledge, attitudes, and practices on catheter-associated UTI prevention: influencing factors in an OB/GYN hospital

**DOI:** 10.3389/fpubh.2025.1517015

**Published:** 2025-02-26

**Authors:** Fei Qu, Yanyu Pang, Mei Wang, Xiaojie Liu, Jing Wang, Li Li

**Affiliations:** ^1^Department of Hospital Infection Management, The Obstetrics and Gynecology Hospital of Fudan University, Shanghai, China; ^2^Department of Nursing, The Obstetrics and Gynecology Hospital, Fudan University, Shanghai, China

**Keywords:** knowledge, attitudes, practices, CAUTI, HAI, cross-sectional study

## Abstract

**Background:**

Catheter-associated urinary tract infection (CAUTI) is a leading cause of hospital-acquired infections globally, with a high prevalence in China, especially in high-risk settings like intensive care and post-operative environments. These infections are influenced by factors such as patient volume, healthcare worker training, and adherence to infection control protocols. Shanghai, as a major healthcare hub, faces unique challenges in CAUTI prevention. Healthcare workers’ knowledge, attitudes, and practices (KAP) play a crucial role in infection control, yet research on factors affecting KAP in obstetrics and gynecology remains limited. The unique patient population and specialized care protocols in these departments present specific challenges, emphasizing the need for deeper insights to enhance prevention strategies.

**Objective:**

The objective was to investigate the KAP scores of healthcare workers in an OB/GYN hospital regarding CAUTI prevention and to identify the factors influencing the scores. The ultimate aim is to provide evidence for improving targeted training programs and infection control measures.

**Methods and participants:**

A cross-sectional study was conducted in an OB/GYN hospital in Shanghai, China. A total of 327 healthcare workers from various departments completed a self-administered questionnaire assessing the KAP scores related to CAUTI prevention. The questionnaire also gathered data on demographic and professional characteristics, CAUTI training frequency, leadership emphasis on infection control, and other relevant factors. Data were analyzed using univariate and multivariate analyses to identify factors significantly influencing KAP scores.

**Results:**

Doctors scored higher than nurses in the knowledge dimension (*p* < 0.001), and increased training frequency was significantly associated with higher knowledge scores (*p for trend* < 0.001). In terms of attitudes, a longer duration of service, more frequent training, and stronger leadership emphasis were all significantly associated with more positive attitudes toward CAUTI prevention (*p for trend* < 0.001). Strong leadership emphasis on CAUTI prevention was also linked to improved practices (*p for trend* < 0.001). The multivariate analysis confirmed that factors such as professional role (doctor vs. nurse), years of service, the role of a clinical instructor, the degree of leadership emphasis, training frequency significantly influenced the scores across various KAP dimensions.

**Conclusion:**

Healthcare workers’ KAP scores toward CAUTI prevention are influenced by factors such as professional role, experience, clinical instruction, leadership emphasis, and training frequency. Tailored, role-specific training and active leadership involvement are essential to improving infection control and reducing CAUTI incidence.

## 1 Introduction

Catheter-associated urinary tract infection (CAUTI) is one of the most prevalent hospital-acquired infections globally, accounting for approximately 40% of all such infections (1). The incidence of CAUTI is closely related to factors such as the duration of catheter use, the method of catheterization, and the underlying health conditions of patients (2, 3). As a common medical device, catheters are widely employed in patients who require urine output monitoring, urinary drainage, or surgical procedures. In obstetric and gynecological surgeries, the use of catheters is particularly prevalent due to the patients’ unique physiological conditions and the necessity for postoperative recovery, which has contributed to an increase in the incidence of CAUTI (4).

In China, CAUTI continues to be a major concern, particularly in high-risk patient populations such as those in intensive care units and post-operative settings (5). The widespread incidence of CAUTI is influenced by various factors, including high patient volumes, variations in healthcare worker training, and inconsistent adherence to infection control protocols. Shanghai, as one of China’s leading medical hubs, plays a crucial role in the country’s healthcare system. The city is home to numerous large medical institutions, including tertiary hospitals specializing in obstetrics and gynecology. These hospitals face unique challenges in infection prevention due to high patient turnover, complex clinical procedures, and varying levels of healthcare worker experience.

The pathogens associated with CAUTI are primarily bacteria, particularly *Escherichia coli*, *Klebsiella* spp., and *Enterococcus* spp. These bacteria are prone to proliferate within the urinary drainage system of catheters, leading to infections. The clinical manifestations of infection can range from asymptomatic bacteriuria to severe urinary tract infections, with symptoms including increased urinary frequency, urgency, dysuria, and fever. In severe cases, CAUTI can lead to complications such as sepsis, which poses significant risks to patient health (2, 6).

In recent years, with the advancement of medical technology and the improvement of nursing standards, many countries have developed guidelines and strategies for the prevention of CAUTI (7, 8). These preventive measures include the rational use of catheters, adherence to strict aseptic protocols, and regular assessments of the necessity for catheterization. However, despite the support of these scientific foundations, inconsistencies between knowledge and practice still occur in actual clinical operations. Numerous studies have indicated that healthcare workers’ knowledge, attitudes, and practices (KAP) regarding CAUTI prevention are critical factors influencing the effectiveness of CAUTI control efforts (9). The KAP levels of healthcare workers directly affect the success rate of infection control measures (10).

This study focuses on understanding the KAP of healthcare workers in Shanghai’s OB/GYN hospital to provide valuable insights into current infection prevention practices. By assessing KAP within this context, the study aims to fill a research gap, offering data that can inform CAUTI prevention strategies both in Shanghai and in similar healthcare institutions across China. The findings are expected to contribute to the development of more effective, targeted prevention and control strategies tailored to the specific needs of high-volume, high-risk healthcare environments. In this context, a thorough analysis of healthcare workers’ KAP levels not only helps identify the existing shortcomings among clinical staff but also provides guidance for hospitals to develop more effective and targeted educational training programs in the prevention of CAUTI. The ultimate aim is to enhance the effectiveness of CAUTI prevention measures and reduce the risk of CAUTI in patients undergoing obstetric and gynecological surgeries.

## 2 Participants and methods

### 2.1 Study population and sample size

The selection criteria for healthcare workers in clinical departments of OB/GYN hospital include staff from the gynecology, obstetrics, and breast surgery departments. The exclusion criteria are as follows: clinical interns, visiting trainees from other institutions, outpatient staff, and medical technicians who are not directly involved in patient care. The sample size for the questionnaire survey was determined based on a cross-sectional design using the following calculation formula:


$$n=\frac{{Z^{2}}_{\alpha /2}pq}{\delta^{2}}$$

With Z_α/2_ set at 1.96 and *p* representing the execution rate of prevention, the initial estimate for this rate was set at 65% based on preliminary pilot testing. Accordingly, *q* was calculated as 1-*p*. The allowable experimental error was set at 0.1*p*, and a two-sided test was employed. Additionally, considering potential loss to follow-up and non-compliance among participants, the sample size was increased by 20% on top of the original calculation to estimate the minimum sample size required for the survey. Ultimately, the target sample size was determined to be 300 participants.

### 2.2 Study design and measurements

The questionnaire was developed based on the “Technical Guidelines for the Prevention and Control of Catheter-Associated Urinary Tract Infections” issued by the Ministry of Health of China, as well as international CAUTI prevention and control guidelines and relevant literature (11–14). During the design process, the practical conditions of the clinical departments at the hospital were integrated, and three rounds of expert consultations and revisions were conducted. The content validity index (CVI) of the questionnaire was 0.95, and the Cronbach’s alpha coefficient was 0.85, confirming the reliability and validity of the questionnaire.

The questionnaire comprises three dimensions: **The knowledge dimension**, which assesses healthcare professionals’ basic understanding of CAUTI prevention and control through 10 T/F questions; **The attitude dimension,** which utilizes a 5-point Likert scale to evaluate attitudes toward preventive measures; **The practice dimension**, which also employs a 5-point Likert scale to measure the preventive behaviors applied in daily clinical practice. These sections offer a comprehensive evaluation of healthcare professionals’ knowledge, attitudes, and behaviors, ensuring the questionnaire’s applicability to clinical settings.

The survey was administered using the professional online survey tool, Wenjuanxing (Changsha Ranxing Information Technology Co., Ltd.),^1^ which generated a QR code for online completion. A convenience sampling approach was employed to survey healthcare professionals from the gynecology, obstetrics, and breast surgery departments who met the inclusion criteria. A standardized training was used to explain the purpose and significance of the survey to the participants. After obtaining informed consent, the researcher presented the QR code during infection control professional training and departmental training sessions. Participants scanned the QR code with their mobile phones and anonymously completed the questionnaire independently. Throughout the study, all data were kept confidential.

### 2.3 Data analysis

The database was constructed using Excel, and statistical analyses were performed with SPSS (v29, IBM, United States). Categorical data were presented as n (%), while continuous data were expressed as $\bar{X}$±s. Differences in KAP scores across various healthcare worker characteristics were compared using t-tests and analysis of variance (ANOVA). If the assumption of homogeneity of variance was not met, the *Welch’s* ANOVA correction was applied. Univariate analysis was initially conducted to identify potential influencing factors, followed by multivariate linear regression analysis with a significance threshold of *p* < 0.10 to further select factors influencing KAP regarding CAUTI prevention. Statistical significance was set at *α* = 0.05, with *p* < 0.05 considered statistically significant.

### 2.4 Ethical considerations

Before conducting the cross-sectional study, an informed consent form and other related documents were reviewed and approved by the ethical clearance committee of the The Obstetrics and Gynecology Hospital of Fudan University (reference no.:2024–128). Additionally, the written informed consent form was obtained from the participants before they responded to the questionnaire.

## 3 Results

A total of 330 questionnaires were collected. After excluding three invalid responses, 327 valid questionnaires were retained for analysis. The questionnaire gathered the baseline characteristics, including age, professional category, clinical department, years of experience, education level, and professional title. Detailed respondent information is presented in Table 1.

**Table 1:** Characteristics of the sample (*n*, %) of participants.

Characteristic	Cat.	No.	%
Gender	Male	24	7.3
	Female	303	92.7
Age (years)	20~	161	49.2
	30~	127	38.8
	40~	33	10.1
	50~	6	1.8
Professional category	Doctor	93	28.4
	Nurse	234	71.6
Clinical department	Gynecology Department	184	56.3
	Obstetrics Department	103	31.5
	Breast Department	12	3.7
	Resident physician	28	8.6
Years of service	<1 year	85	26.0
	5–10 year	120	36.7
	>10 year	122	37.3
Education degree	Below bachelor	57	17.4
	Bachelor	199	60.9
	Master/doctor	71	21.7
Professional title	Junior	238	72.8
	Intermediate	80	24.5
	Senior	9	2.8
Clinical instructor	No	271	82.9
	Yes	56	17.1
Training frequency	None	88	26.9
	Once	91	27.8
	Twice	39	11.9
	≥3 Times	109	33.3
Degree of leadership emphasis	Not committed	0	0.0
	Neutral	10	3.1
	Committed	78	23.9
	Highly committed	239	73.1

In this study, we conducted a comprehensive analysis of the KAP scores related to the prevention of CAUTI among healthcare workers in an OB/GYN hospital. Various influencing factors were explored, and the results demonstrated that gender, age, years of service, professional category, educational level, clinical department, frequency of training, and the level of leadership emphasis all had varying degrees of impact on the KAP scores of healthcare personnel (seen Table 2).

**Table 2:** Knowledge, attitude, and practice scores on the CAUTI questionnaire.

Characteristics	Cat.	Knowledge		Attitude		Practice	
		Score	*p*	Score	*p*	Score	*p*
Gender	Male	7.58 ± 1.10	0.028	21.92 ± 2.10	<0.001	35.58 ± 4.18	0.155
	Female	7.03 ± 1.18		22.91 ± 2.23		36.65 ± 3.49	
Age (years)	20~	7.11 ± 1.20	0.789	22.27 ± 2.33	0.001^*^	36.29 ± 3.71	0.559
	30~	7.08 ± 1.17		23.36 ± 2.11		36.85 ± 3.40	
	40~	6.88 ± 1.17		23.30 ± 2.01		36.79 ± 3.53	
	50~	7.17 ± 1.17		24.33 ± 1.63		37.17 ± 2.48	
Professional category	Doctor	7.42 ± 1.01	<0.001	22.29 ± 2.21	0.005	36.27 ± 4.10	0.371
	Nurse	6.94 ± 1.22		23.06 ± 2.21		36.70 ± 3.31	
Clinical department	Gynecology	7.03 ± 1.14	0.464	22.82 ± 2.18	0.055	36.26 ± 3.63	<0.001
	Obstetrics	7.17 ± 1.25		22.94 ± 2.28		37.19 ± 2.79	
	Breast	7.42 ± 1.51		24.08 ± 1.31		39.17 ± 1.59	
	Resident	6.89 ± 1.03		22.04 ± 2.53		35.29 ± 5.05	
Years of service	<1 year	7.25 ± 1.13	0.289	22.09 ± 2.20	<0.001^*^	36.11 ± 3.71	0.368
	5 ~ 10 year	7.02 ± 1.18		22.51 ± 2.45		36.73 ± 3.63	
	>10 year	7.01 ± 1.21		23.68 ± 1.72		36.75 ± 3.35	
Education degree	Below bachelor	6.91 ± 1.29	0.012^*^	22.96 ± 2.25	0.282	36.81 ± 2.86	0.761
	Bachelor	6.99 ± 1.18		22.93 ± 2.21		36.59 ± 3.50	
	Master/doctor	7.44 ± 1.02		22.46 ± 2.27		36.35 ± 4.17	
Professional title	Junior	7.08 ± 1.17	0.775	22.69 ± 2.27	0.132	36.51 ± 3.52	0.858
	Intermediate	7.04 ± 1.22		23.18 ± 2.14		36.75 ± 3.62	
	Senior	7.33 ± 1.12		23.67 ± 1.94		36.78 ± 4.02	
Clinical instructor	No	7.06 ± 1.17	0.544	22.62 ± 2.31	<0.001	36.38 ± 3.67	0.010
	Yes	7.16 ± 1.26		23.88 ± 1.44		37.52 ± 2.76	
Training frequency	None	6.83 ± 1.33	0.017^*^	22.20 ± 2.27	0.008^*^	35.95 ± 3.85	0.281
	Once	6.96 ± 1.14		22.82 ± 2.00		36.74 ± 3.09	
	Twice	7.18 ± 1.10		23.05 ± 2.18		37.00 ± 3.13	
	≥3 Times	7.33 ± 1.06		23.28 ± 2.32		36.79 ± 3.77	
Degree of leadership emphasis	Neutral	7.40 ± 0.84	0.277	20.10 ± 1.45	<0.001^*^	33.20 ± 5.55	0.001^*^
	Committed	7.22 ± 1.09		21.54 ± 2.39		35.32 ± 3.62	
	Highly committed	7.01 ± 1.22		23.38 ± 1.94		37.13 ± 3.25	

*Refer to *p* for trend.

Within the knowledge domain, there were notable gender differences. Male healthcare workers had significantly higher knowledge scores (7.58 ± 1.10) compared to their female counterparts (7.03 ± 1.18), with a statistically significant difference (*p* = 0.028). Regarding professional categories, doctors had significantly higher knowledge scores (7.42 ± 1.01) than nurses (6.94 ± 1.22), with *p* < 0.001, indicating that doctors had a better grasp of CAUTI-related knowledge. The frequency of CAUTI training was positively correlated with knowledge scores. Participants who had not received any training had the lowest scores (6.83 ± 1.33), while those who had undergone training three or more times had the highest scores (7.33 ± 1.06), with a statistically significant difference (*p for trend* = 0.017). This suggests that more frequent training leads to better knowledge retention. Additionally, educational background played a significant role, with healthcare workers holding higher degrees, such as a master’s or doctorate, scoring the highest (7.44 ± 1.02). The *p for trend* was 0.012, indicating a significant positive correlation between educational attainment and knowledge scores (Table 3).

**Table 3:** The correct response rate in the knowledge dimension of the CAUTI scale.

Serial number	Items	n (%)
1	Postoperative catheter removal in surgical patients typically occurs between 7 and 14 days	260 (79.5%)
2	Maintain a closed, patent drainage system; clamp the catheter during mobilization or transport	213 (65.1%)
3	Assess the necessity of catheterization daily and remove it as early as possible	312 (95.4%)
4	Send urine for culture if a urinary tract infection is suspected.	323 (98.8%)
5	Replace the catheter if a urinary tract infection occurs	242 (74.0%)
6	Use separate containers for each patient’s urine bag	290 (88.7%)
7	Daily disinfect the urethral meatus with povidone-iodine for patients with in dwelling catheters	46 (14.1%)
8	Store urine culture specimens at room temperature for up to 24 h if delayed	271 (82.9%)
9	Do not treat asymptomatic bacteriuria in catheterized patients unless they are high-risk, like pregnant women or those facing mucosal bleeding during urological procedures.	269 (82.3%)
10	Routinely replace catheters in long-term catheterized patients to prevent infection	87 (26.6%)

Regarding the attitude aspect, female healthcare workers had significantly higher attitude scores (22.91 ± 2.23) compared to males (21.92 ± 2.10), with a statistically significant difference (*p* < 0.001). Attitude scores increased significantly with age, with the highest scores in the 50 and above age group (24.33 ± 1.63). The *p for trend* was 0.001, indicating a significant positive correlation between age and attitudes toward CAUTI prevention. A similar trend was observed with years of service, where longer service corresponded to higher scores. The highest average score (23.68 ± 1.72) was achieved by those with over 10 years of experience (*p for trend* < 0.001). Nurses had slightly higher attitude scores (23.06 ± 2.21) than doctors (22.29 ± 2.21), with the difference being statistically significant (*p* = 0.005). This suggests that nurses tend to have a more positive attitude toward CAUTI prevention compared to doctors. The emphasis placed by leadership on CAUTI prevention significantly influenced healthcare workers’ attitudes. When leadership demonstrated a high level of commitment, attitude scores were the highest (23.38 ± 1.94), with a *p for trend* (<0.001), highlighting the importance of leadership in fostering positive attitudes toward infection prevention.

In terms of practice, healthcare workers in the breast department achieved notably higher practice scores (39.17 ± 1.59) than those in other departments, with a statistically significant result (*p* < 0.001). This finding illustrates the marked disparity in practice levels between departments. Clinical instructor involvement had a significant impact, as workers with experience in clinical instruction scored higher (37.52 ± 2.76) compared to those without such experience (36.38 ± 3.67), with a statistically meaningful difference (*p* = 0.010). Leadership commitment to CAUTI prevention was strongly linked to better practice outcomes, with the trend showing a significant positive correlation (*p for trend* = 0.001). This result highlights the important influence of leadership in driving improved infection prevention practices among healthcare personnel (Table 4).

**Table 4:** Multiple linear regression analysis of independent influences under different dimensions.

Dimensions	Characteristics	*β*	SE	*t*	*p*	95% CI	
						Lower	Upper
Knowledge	Nurse vs. Doctor	−0.549	0.141	−3.899	<0.001	−0.826	−0.272
	CAUTI training frequency	0.197	0.053	3.742	<0.001	0.093	0.301
Attitude	Years of service	0.419	0.152	2.753	0.006	0.119	0.718
	Clinical instructor	1.090	0.300	3.632	<0.001	0.499	1.680
	Nurse vs. Doctor	0.360	0.269	1.337	0.182	−0.170	0.891
	Department						
	Gynecology	Reference					
	Obstetrics	0.091	0.246	0.370	0.711	−0.393	0.576
	Breast	1.122	0.591	1.898	0.059	−0.041	2.284
	Resident	−0.242	0.404	−0.599	0.549	−1.038	0.553
	Degree of leadership emphasis						
	Neutral	Reference					
	Committed	1.144	0.664	1.723	0.086	−0.162	2.451
	Highly committed	2.651	0.649	4.087	<0.001	1.375	3.927
Practice	Department						
	Gynecology	Reference					
	Obstetrics	1.024	0.413	2.478	0.014	0.211	1.838
	Breast	2.888	0.999	2.892	0.004	0.924	4.853
	Resident	−0.562	0.685	−0.822	0.412	−1.909	0.785
	Clinical instructor	0.939	0.494	1.900	0.058	0.033	1.910
	Degree of leadership emphasis						
	Neutral	Reference					
	Committed	3.547	1.087	3.265	0.001	1.409	5.685
	Highly committed	1.792	1.128	1.588	0.113	−0.428	4.011

Interestingly, within the 10 T/F questions in the knowledge dimension, we found that several items had a correct response rate of less than 30%. These questions likely reflect areas of confusion among frontline healthcare workers, indicating where future training efforts should be focused. Specifically: **Question 7**: “Daily disinfect the urethral meatus with povidone-iodine for patients with in dwelling catheters,” the correct response rate of only 14.1%. This suggests that there may be significant misunderstanding or unclear awareness among healthcare workers regarding how to maintain a sterile environment around catheters. Training should place more emphasis on this topic, helping staff understand the importance of maintaining cleanliness around the catheter site to prevent CAUTI, and clarifying that saline solution is sufficient for daily maintenance in most cases. **Question 10**: “Routinely replace catheters in long-term catheterized patients to prevent infection,” it had a correct response rate of 26.6%. This may indicate a lack of understanding regarding when catheter replacement is necessary to prevent infection. It is recommended that training focus on management strategies for long-term indwelling catheters, particularly highlighting when and why catheter replacement is required to prevent infections (Table 3).

Through multiple regression analysis, we found that doctors had significantly higher knowledge scores compared to nurses (*p* < 0.001), and more frequent CAUTI-related training was associated with an increase in knowledge scores (*p* < 0.001). In the attitude dimension, healthcare workers with longer years of service and those serving as clinical instructors had significantly higher attitude scores (*p* = 0.006 and *p* < 0.001, respectively). Additionally, a strong emphasis on CAUTI prevention from leadership led to notable improvements in both attitude (*p* < 0.001) and practice scores (*p* = 0.001). Workers in the breast department had significantly higher practice scores compared to those in gynecology and obstetrics (*p* = 0.004) (Table 4).

## 4 Discussion

In this study, we conducted an in-depth analysis of healthcare workers’ knowledge, attitudes, and practices (KAP) related to CAUTI prevention in an OB/GYN hospital. The results showed that the KAP of healthcare workers was influenced by various factors, including gender, professional category, years of service, educational level, department, frequency of training, and the emphasis placed on CAUTI prevention by leadership. These findings not only highlight key factors in infection control within hospitals but also align with current international research, further enhancing our understanding of KAP disparities in CAUTI prevention.

Our study found that doctors had significantly higher knowledge scores than nurses (*p* < 0.001), and the frequency of CAUTI-related training was positively correlated with knowledge scores (*p* = 0.017). This difference may be attributed to the more comprehensive and specialized training doctors receive, particularly in infection prevention and control. For instance, a systematic review has shown that doctors possess better infection control knowledge than other healthcare workers, largely due to the structured education they undergo and their more frequent exposure to complex clinical cases in their daily practice (15). Additionally, multiple international studies have demonstrated that targeted infection control training effectively improves healthcare workers’ knowledge and practice capabilities (9, 16, 17). Our data corroborate these findings, suggesting that increased training frequency significantly enhances healthcare workers’ understanding of CAUTI prevention. Therefore, the positive correlation between training frequency and knowledge levels is well-supported. Regarding educational level, we found that healthcare workers with higher levels of education, particularly those with master’s or doctoral degrees, performed better in the knowledge dimension (*p for trend* = 0.012). This finding showed that healthcare workers with higher education tend to handle complex infection control issues more competently (9). Therefore, raising the overall education level of healthcare workers or providing more training opportunities for those with lower education levels can effectively improve infection control across the board.

In terms of attitude, female healthcare workers scored significantly higher than male workers (*p* < 0.001), which may be explained by gender roles in health behaviors. Female healthcare workers are often more attentive to patient care details, and this gender difference is particularly notable in infection prevention attitudes. Previous studies have indicated that women are more likely to actively learn and adopt infection control measures (15, 18). Moreover, healthcare workers with longer years of service had more positive attitudes, as did those with clinical instructor experience, which indicate that longer clinical experience and greater responsibility result in a stronger sense of accountability and attention to infection control, because they better understand its long-term benefits and are more confident in the effectiveness of the measures (19, 20). Leadership’s emphasis on CAUTI prevention had a significant impact on healthcare workers’ attitudes, with higher leadership commitment correlating with significantly improved attitude scores. It highlights the role of leadership in shaping healthcare workers’ perceptions and behaviors related to infection control (21, 22). The survey noted that leadership involvement and support are critical factors in the successful implementation of hospital infection control measures (23).

Regarding practice, we found that healthcare workers in the breast department scored significantly higher in practice compared to those in gynecology and obstetrics (*p* < 0.001). This difference may be related to the varying clinical practice characteristics across departments, like clinical workflows, patient populations, and the emphasis on infection control within each department. For instance, breast department patients typically present with lower infection risks, which may make it easier for healthcare workers to implement infection prevention measures. In contrast, gynecology and obstetrics departments face higher infection risks and greater workloads, which may challenge staff in fully adhering to infection control standards.

Some researchers have similarly shown that different clinical departments often have varying levels of infection control performance, with some departments implementing stricter prevention measures due to higher infection risks and turnover of beds (time). Moreover, leadership’s emphasis on infection control also had a significant impact on practice scores, with higher leadership commitment leading to better practical outcomes. This finding aligns with international studies that emphasize the role of leadership in driving improved infection control practices. Leadership support and clear infection control policies have been shown to be key drivers in improving healthcare workers’ infection control practices.

One of the most significant findings from this study is the relationship between CAUTI training frequency and improved knowledge and practice scores. The data clearly indicate that more frequent training leads to better knowledge retention and the practical implementation of CAUTI prevention measures. This is consistent with international research, which shows that regular and structured training is essential for improving the capabilities of frontline healthcare workers (6, 24, 25). Additionally, international studies have demonstrated that combining simulation-based training with hands-on clinical education can effectively enhance compliance with infection control protocols (26, 27).

Our study also observed some unique phenomena. Within the context of obstetrics and gynecology, the practice score differences were more pronounced. Healthcare workers in the breast department showed superior behavior in practice compared to those in gynecology and obstetrics. This difference likely reflects variations in patient infection risks and work environments across departments. In other studies, differences in practice scores between departments were smaller, which may be attributable to differences in study settings or participant selection.

Due to the use of convenience sampling, the sample may not represent the broader population of healthcare professionals. Future studies could consider employing random sampling methods and extending the research to other hospitals for comparison, thereby enhancing the external validity and generalizability of the findings. While our study provides valuable insights into the KAP of healthcare workers regarding CAUTI prevention, we acknowledge the limitations associated with the use of self-administered surveys. Self-reporting, while convenient and cost-effective, may be prone to social desirability bias, where participants may report behaviors, they believe are expected or deemed socially acceptable. Furthermore, observational methods should also be incorporated in future research to directly assess the behaviors of healthcare workers, providing a more accurate measure of infection prevention practices.

## 5 Conclusion

This study demonstrates that healthcare workers’ knowledge, attitudes, and practices in preventing CAUTI are influenced by various factors, including gender, professional category, years of service, educational level, department, and frequency of training. Notably, leadership’s emphasis on CAUTI prevention and whether healthcare workers have received relevant training significantly impact their attitudes and practical capabilities in CAUTI prevention. Therefore, hospitals should prioritize focused training and education on CAUTI prevention, especially for nurses and those with fewer years of service, to enhance their infection control capabilities in clinical practice. Additionally, the active involvement and commitment of leadership in infection control are crucial for improving overall prevention outcomes. It is recommended that hospital management further strengthen support for CAUTI prevention efforts by implementing comprehensive training and management policies, thereby optimizing frontline infection control and reducing the incidence of CAUTI.

## Data Availability

The raw data supporting the conclusions of this article will be made available by the authors, without undue reservation.

## Footnotes

^1^ https://www.wjx.cn/
